# Comparative Study of the Mechanisms Underlying the Effects of Prohexadione-Calcium and Gibberellin on the Morphogenesis and Carbon Metabolism of Rice Seedlings Under NaCl Stress

**DOI:** 10.3390/plants14081240

**Published:** 2025-04-18

**Authors:** Meiling Liu, Naijie Feng, Dianfeng Zheng, Fengyan Meng

**Affiliations:** 1 College of Coastal Agriculture Sciences, Guangdong Ocean University, Zhanjiang 524000, China; lml19960416@126.com (M.L.); 13598721953@163.com (F.M.); 2 Shenzhen Research Institute of Guangdong Ocean University, Shenzhen 518000, China

**Keywords:** rice, NaCl stress, prohexadione–calcium, gibberellin, carbon metabolism

## Abstract

NaCl stress is one of the most serious forms of salt stress. Prohexadione–calcium (EA) is a plant growth regulator, and gibberellin (GA) is a plant hormone that regulates various plant developmental processes. In this experiment, Guanghong 3 and Huang Huazhan served as experimental rice (*Overza sativa* L.) varieties to study the effects of EA and GA on the growth of rice seedlings. The results revealed that NaCl treatment significantly inhibited plant growth and destroyed the balance of the carbon metabolism. The inhibition effect of NaCl stress on the growth and physiological metabolism of rice seedlings was alleviated by EA and GA, but the effects of EA and GA were slightly different. Compared with the NaCl treatment, the EA and GA treatments significantly increased the net photosynthetic rate, stem base width, and dry matter accumulation but had opposite effects on the plant height, with the GA treatment significantly increasing the plant height of rice seedlings. The EA treatment was superior to the GA treatment in improving the metabolic pathway efficiency of sucrose and starch in the leaves of rice seedlings. The soluble sugar content, sucrose content, fructose content, sucrose synthase activity, sucrose phosphate synthase activity, α-amylase activity, β-amylase activity, and starch phosphorylase activity increased with increasing NaCl stress time, and the changes in the starch content and acid invertase activity were the opposite. The max/min values were reached on the 13th day of NaCl stress.

## 1. Introduction

Studies claim that soil salinization is associated with secondary salinization caused by human activities and that half of the world’s available arable land will be under salt stress by 2050 [1]. Sodium chloride is one of the main salts enriched on the soil surface [2]. Photosynthesis efficiency plays an important role in plant productivity, and the photosynthetic carbon assimilation process of carbon-3 plants is affected by the water status of the plant and various environmental factors [3]. Salt stress has serious effects on plant growth, affecting the photosynthesis, osmotic regulation, and carbon metabolism processes [4,5]. Under salt stress, plant metabolism is regulated by carbohydrates in order to maintain basic growth [6]. Therefore, carbohydrates play a key role in the defence mechanism of plants under abiotic stress [7]. Previous studies have found that overexpression of gene clusters involved in polyamine transport leads to greater phenolamide accumulation and diversity, thereby enhancing crop salt tolerance [8].

Rice (*Overza sativa* L.) is classed as a crop sensitive to salt [9]. The seedling stage is the period during which the plant’s abiotic stress resistance is the weakest [10]. Thus, rice seedlings are particularly sensitive to salt [1]. Salt stress will change the biological processes of seedlings during growth and development, including their penetration stability, photosynthesis, and carbohydrate metabolism [11]. Plants regulate the photosynthesis and carbon metabolism by accumulating sucrose and other substances under stress [12]. A reduction in the starch content can occur due to carbon limitation as a result of a reduction in the photosynthesis rate under salt stress [13]. Increasing the salt resistance of rice seedlings can provide a basis for enhanced salt resistance throughout the entire life cycle of the rice plant. As such, research investigating methods of enhancing the salt tolerance of rice seedlings is a critical focus of the field.

Plant growth regulators affect the growth and development of organs or tissue by changing the physiological metabolism process of plants [14]. Plant growth regulators are widely used in agricultural crops as a means of crop improvement. Prohexadione–calcium (EA) is a type of plant growth inhibitor. By inhibiting the plant’s height, it enhances the plant’s resistance [15]. Studies have shown that EA reduces plant height by preventing the conversion of gibberellin GA_20_ to gibberellin GA_1_ [16]. EA has been shown to have good morphological inhibitory effects on plants such as eggplant [17] and soybean [18].

Gibberellin (GA) is one of the most widely used plant growth regulators [19] and has a positive effect on promoting plant height and nutrient absorption [20]. Previous studies claimed that exogenous GA3 significantly enhanced crop germination and improved crop tolerance to salt stress [21]. In this experiment, the effects of NaCl stress on the morphogenesis, photosynthesis, and sucrose–starch catabolism or synthesis pathways of rice seedlings by prohexadione–calcium and gibberellin leaf spraying were comparatively investigated to assess the differences in the regulation of carbohydrate metabolism pathways between prohexadione–calcium and gibberellin. These results can be used as a reference to study the effect of NaCl stress on the photosynthesis and carbohydrate metabolism of rice seedlings and to understand the effect of prohexadione–calcium and gibberellin in alleviating NaCl stress on rice seedlings.

## 2. Materials and Methods

### 2.1. Plant Materials and Growth Conditions

The testing was conducted in the greenhouses of the Guangdong University of Ocean University from November 2021 to October 2022. The test species and regulators were provided by the Binhai Academy of the Agricultural College of Guangdong Ocean University. This experiment was performed on two rice varieties: Guanghong 3 (No. 3) and Huang Huazhan (HHz). We selected rice seeds that were full and intact, disinfected them with 3% hydrogen peroxide for 15 min, and rinsed them with distilled water five times. The potting method was as follows (day/night temperature of 25 ± 2 °C/20 ± 2 °C). Rice seeds of uniform dewlap size were spotted in non-porous plastic pots (upper diameter, lower diameter, and height = 15 cm, 11.5 cm, and 14.7 cm, respectively) with a cultivation substrate of brick red soil–vermiculite = 3:1. Each rice variety was treated with six different treatments: (1) CK (control): water treatment; (2) EA: 100 mg·L^−1^ EA sprayed on the leaves; (3) GA: 1 mg·L^−1^ GA sprayed on the leaves; (4) N: 50 mM NaCl treatment; (5) EAN: 100 mg·L^−1^ EA sprayed on the leaves + 50 mM NaCl treatment; and (6) GAN: 1 mg·L^−1^ GA sprayed on the leaves + 50 mM NaCl treatment. When rice seedlings grew to three leaves and one heart, 100 mg·L^−1^ EA and 1 mg·L^−1^ GA leaf spray were applied to the regulator leaf spray until the leaf surface was moistened. Salt treatment was carried out after 24 h of conditioner treatment; i.e., 50 mM NaCl (*w*/*w*) [22] was applied according to the soil volume, retaining a 2 cm water layer. On the 4th, 7th, 10th, and 13th days after NaCl treatment, the morphological and carbon metabolism indices were measured. Salt concentration was maintained daily by real-time salinity monitoring with a soil salinometer(soil salinometer: STEPS, Nuremberg, Germany, PNT Combi5000). The EA concentration and GA concentration were determined in the pre-test via pre-experimentation.

### 2.2. Measurement of Morphological Indicators

On the 4th, 7th, 10th, and 13th days after NaCl stress, 20 rice seedlings with the same growth vigor and size were selected, and the height of each plant was measured with a ruler. The rice seedling stem base width was measured using vernier callipers, accurate to two decimal places. The above-ground parts of rice seedlings were washed, blotted dry, placed in paper bags, put into an oven at 105 °C, and killed until the mass became constant. Their dry weight was determined using an electronic analytical balance.

### 2.3. Gas Exchange Parameter Measurement

The net photosynthetic rate (Pn) of the fully expanded leaves (widest position in the middle of the leaf) of the rice seedlings was measured using a LI-6800 portable photosynthetic apparatus (6800-01A, LI-COR Biosciences, Lincoln, NE, USA) on a clear day between 9:00 a.m. and 11:00 a.m. The indoor light intensity, CO_2_, and air temperature were set in accordance with the environmental conditions within the plot.

### 2.4. Determination of Carbon Metabolic Products and Related Enzyme Activity

We determined the soluble sugar, sucrose, and fructose contents of rice leaves by referring to the experimental method of Tian et al. [23].

Based on the methods of previous studies, starch content [2], sucrose synthase (SS) [2], amylase activity [4], sucrose phosphate synthase (SPS) activity [2], acid convertase (AI) [24], and starch phosphorylase activity [25] were determined in rice leaves.

### 2.5. Statistical Analysis

Each treatment was replicated three times independently, and data for each parameter are described as mean ± SE (standard error). Analysis of variance and Duncan’s multiple range test were performed for each parameter using the statistical software SPSS 24 with *p* < 0.05. Factor analysis (two-way ANOVA) was used to test for NaCl treatment time, NaCl treatment (N), “prohexadione–calcium + NaCl treatment” (EAN) level, “gibberellin + NaCl treatment” (GAN) level, and different main effects of interactions. Graphs were plotted using Origin2021 software, and * in the graphs indicates differences at the 0.05 level and ** indicates differences at the 0.01 level.

## 3. Results

### 3.1. Changes in Morphological Establishment of Rice Seedlings

**Figure 1:**
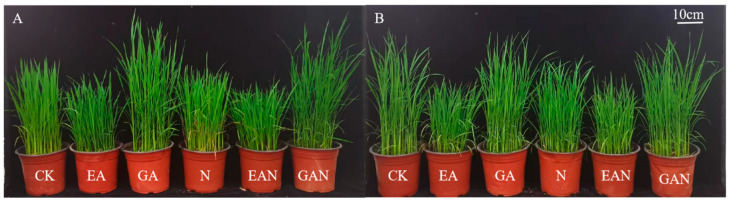
Effects of NaCl stress, prohexadione–calcium, and gibberellin treatment on the growth of rice seedings. Figure is taken from 13 days after NaCl treatment: (**A**) No. 3 rice variety; (**B**) HHz rice variety. CK, clear water treatment; EA, 100 mg·L^−1^ prohexadione–calcium leaf spray; GA, 1 mg·L^−1^ gibberellin leaf spray; N, 50 mmol·L^−1^ NaCl treatment; EAN, 100 mg·L^−1^ prohexadione–calcium leaf spray + 50 mmol·L^−1^ NaCl treatment; GAN, 1 mg·L^−1^ gibberellin leaf spray + 50 mmol·L^−1^ NaCl treatment.

**Figure 2:**
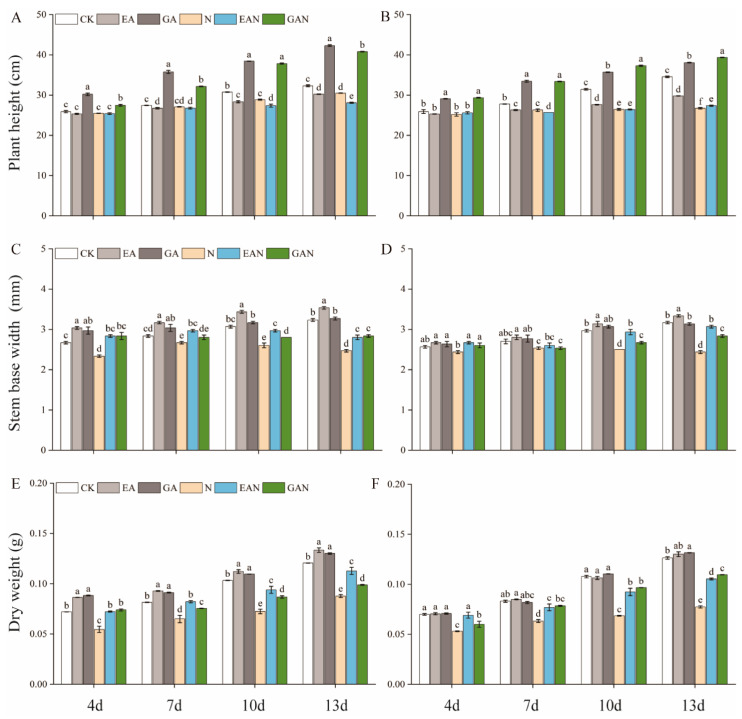
The impact of prohexadione–calcium and gibberellin on the formation of rice seedlings under NaCl stress. (**A**,**C**,**E**) refer to No. 3; (**B**,**D**,**F**) refer to HHz. Abbreviations: CK, control (normal water); EA, foliar prohexadione–calcium application; GA, foliar gibberellin application; N, NaCl stress; EAN, foliar prohexadione–calcium application + NaCl stress; GAN, foliar gibberellin application + NaCl stress. Values represent the mean ± SE (*n* = 3), and different lowercase letters indicate significant differences according to Duncan’s test.

NaCl processing significantly inhibited the growth of No. 3 and HHz seedlings (Figure 1). The plant height of No. 3 and HHz GAN treatments were significantly higher than the N treatment, with a significant increase of 3.77% and 47.32%, respectively, at the 13th day of NaCl treatment compared to the N treatment. EA had a significant inhibitory effect on the height of rice seedlings. The plant height of No. 3 under the EAN treatment was 7.87% lower than that under the N treatment on day 13, while the height of HHz under the EAN treatment was 2.50% higher on day 13 than that of the N treatment (Figure 2A,B). EA and GA treatments had a better promotion effect on the increase in the stem base width and dry weight of rice seedlings under NaCl stress. The stem base width of No. 3 under the EAN and GAN treatments significantly increased by 13.51% and 14.86%, respectively, and the dry weight significantly increased by 28.47% and 12.53%, respectively, compared to the N treatment on the 13th day of NaCl treatment (Figure 2C,E). For the HHz variety, the basal stem width increased by 26.03% and 16.44%, and the dry weight increased by 36.18% and 41.60% in the EAN and GAN treatments, respectively, on day 13 of NaCl treatment, as compared with N treatment alone (Figure 2D,F).

### 3.2. Changes in the Net Photosynthetic Rate (Pn)

**Figure 3:**
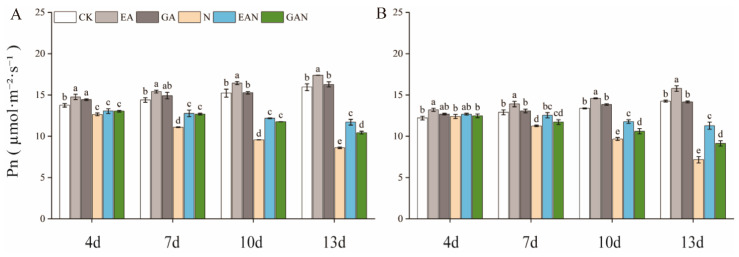
Effect of prohexadione–calcium and gibberellin on the net photosynthetic rate (Pn) of rice seedlings under NaCl stress: (**A**) No. 3; (**B**) HHz. Abbreviations: CK, control (normal water); EA, foliar prohexadione–calcium application; GA, foliar gibberellin application; N, NaCl stress; EAN, foliar prohexadione–calcium application + NaCl stress; GAN, foliar gibberellin application + NaCl stress. Values represent the mean ± SE (*n* = 3), and different lowercase letters indicate significant differences according to Duncan’s test.

Prolonged NaCl stress resulted in a gradual decrease in the net photosynthetic rate of rice seedlings (Figure 3). The inhibition of the Pn of rice seedlings by NaCl stress was alleviated by EA and GA treatment. From the 10th day to the 13th day, the Pn increased by 27.20–36.19% and 21.39–22.88% in the EAN and GAN treatment groups, respectively, for No. 3; while for HHz, these increases were 21.79–57.58% and 9.72–27.54%, respectively (Figure 3A,B).

### 3.3. Changes in the Carbohydrate Content

**Figure 4:**
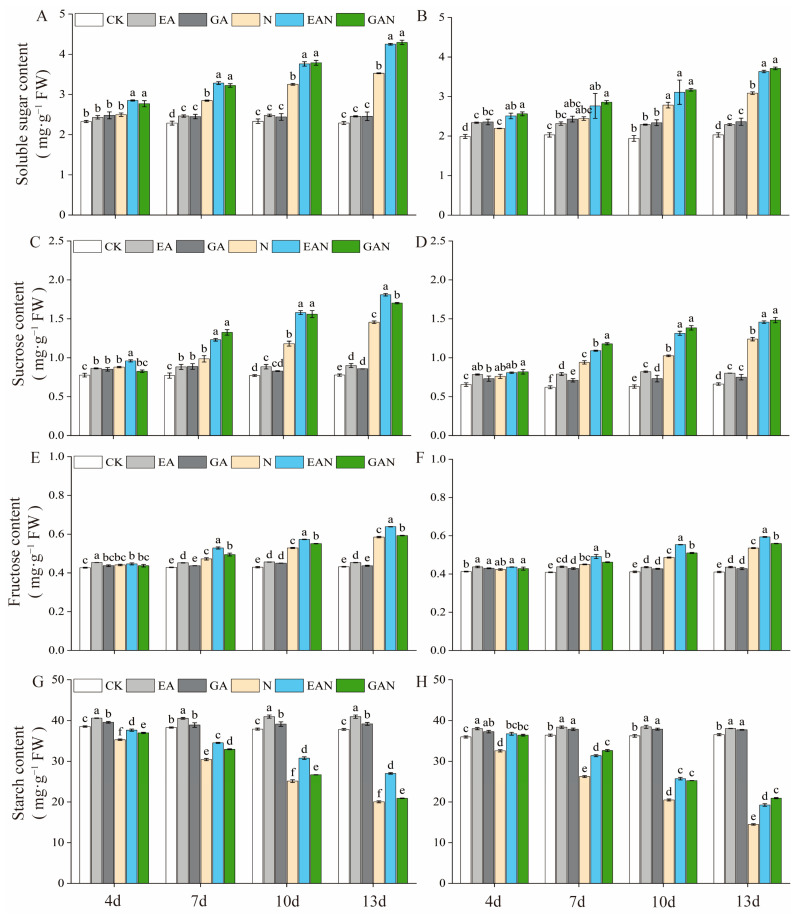
Effect of prohexadione–calcium and gibberellin on the carbohydrate content of the leaves of rice seedlings under NaCl stress. (**A**,**C**,**E**,**G**) refer to No. 3; (**B**,**D**,**F**,**H**) refer to HHz. Abbreviations: CK, control (normal water); EA, foliar prohexadione–calcium application; GA, foliar gibberellin application; N, NaCl stress; EAN, foliar prohexadione–calcium application + NaCl stress; GAN, foliar gibberellin application + NaCl stress. Values represent the mean ± SE (*n* = 3), and different lowercase letters indicate significant differences according to Duncan’s test.

The contents of soluble sugar, sucrose, and fructose in the leaves of rice seedlings increased with increasing NaCl treatment time, while the content of starch decreased (Figure 4). Under NaCl stress, EA and GA decreased the osmotic potential of rice seedlings by further increasing the contents of osmoregulatory substances. The soluble sugar, sucrose, and fructose contents of No. 3 under the EAN and GAN treatments reached maximum values on the 13th day of NaCl treatment; compared with the N treatment, they were significantly increased by 20.51% and 21.74%, 24.31% and 16.91%, and 9.15% and 1.35%, respectively. For HHz, these increases were 17.71% and 20.30%, 17.70% and 19.73%, and 10.86% and 4.42% for soluble sugar, sucrose, and fructose, respectively, with all increases reaching statistical significance (Figure 4A–F). The starch content in the leaves of rice seedlings was the smallest on the 13th day after NaCl treatment. The starch content of rice seedling leaves was the smallest at the 13th day of NaCl treatment; the EAN treatment of No. 3 was more effective in alleviating the decrease in starch content, and the GAN treatment of HHz was more effective in accomplishing the same thing. On the 13th day of NaCl stress, for the EAN and GAN treatments on the No. 3 variety, the starch content increased by 34.83% and 4.30%; while for the HHz variety, the EAN and GAN treatments increased the starch content by 32.95% and 44.61%, respectively (Figure 4G,H).

### 3.4. Changes in the Sucrose Metabolic Pathway

**Figure 5:**
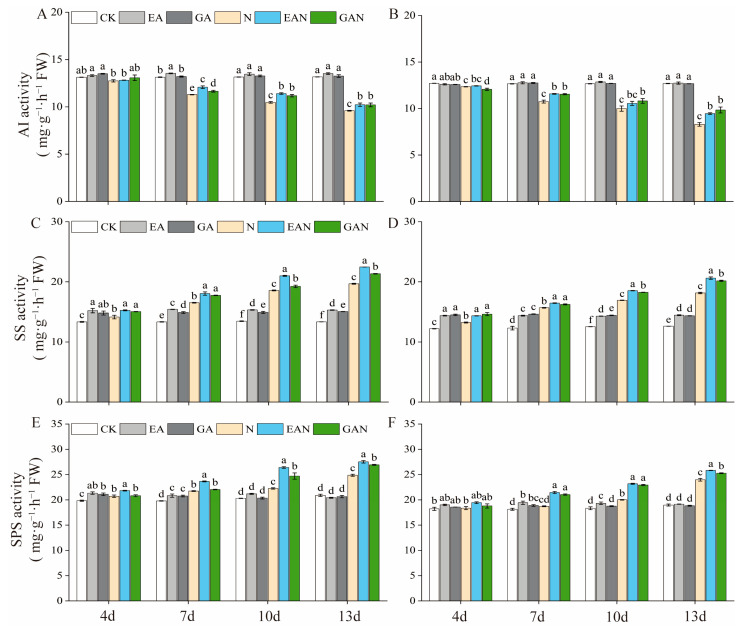
The effects of prohexadione–calcium and gibberellin treatments on the activities of enzymes involved in sucrose synthesis or decomposition in rice seedlings under NaCl stress. (**A**,**C**,**E**) refer to No. 3; (**B**,**D**,**F**) refer to HHz. Abbreviations: CK, control (normal water); EA, foliar prohexadione–calcium application; GA, foliar gibberellin application; N, NaCl stress; EAN, foliar prohexadione–calcium application + NaCl stress; GAN, foliar gibberellin application + NaCl stress. Values represent the mean ± SE (*n* = 3), and different lowercase letters indicate significant differences according to Duncan’s test.

The activities of SS and SPS in the leaves of rice seedlings increased with increasing NaCl treatment time, while the activity of AI decreased (Figure 5). Under NaCl stress, AI activity decreased, while SS and SPS activities increased. EA treatment was superior to GA treatment in promoting the AI, SS, and SPS activities under NaCl stress (Figure 5). On day 13, the AI, SS, and SPS activities of the No. 3 variety under the EAN and GAN treatments were significantly increased by 6.56% and 6.41%, 14.18% and 8.45%, and 10.71% and 8.30%, respectively, compared with the N treatment (Figure 5A,C,E). On day 13, the AI, SS, and SPS activities of the HHz variety under the EAN and GAN treatments were significantly increased by 14.02% and 18.67%, 13.62% and 11.14%, and 7.67% and 5.41%, respectively, compared with the N treatment (Figure 5B,D,F).

### 3.5. Changes in the Starch Metabolism Pathway

**Figure 6:**
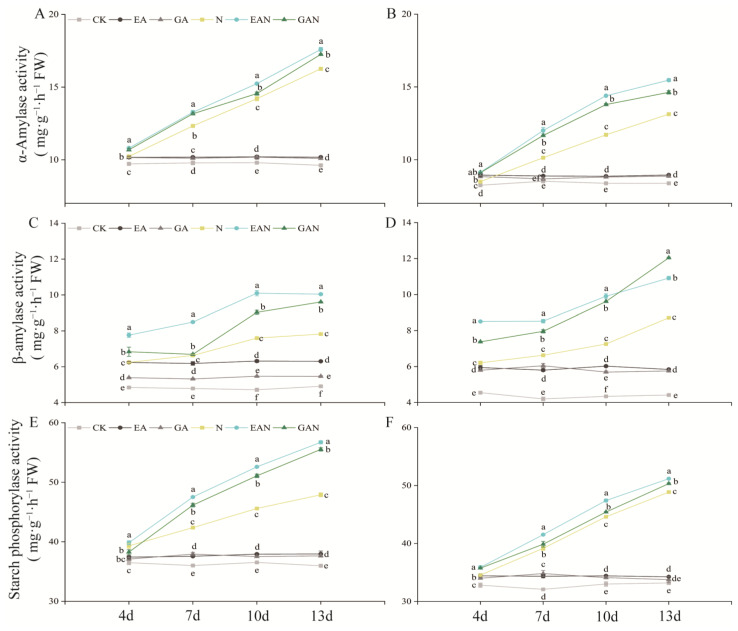
Effects of prohexadione–calcium and gibberellin on the activities of enzymes involved in starch synthesis or decomposition in rice seedlings under NaCl stress. (**A**,**C**,**E**) refer to No. 3; (**B**,**D**,**F**) refer to HHz. Abbreviations: CK, control (normal water); EA, foliar prohexadione–calcium application; GA, foliar gibberellin application; N, NaCl stress; EAN, foliar prohexadione–calcium application + NaCl stress; GAN, foliar gibberellin application + NaCl stress. Values represent the mean ± SE (*n* = 3), and different lowercase letters indicate significant differences according to Duncan’s test.

The activities of α-amylase, β-amylase, and starch phosphorylase activities in the leaves of rice seedlings increased with increasing NaCl treatment time (Figure 6). NaCl stress resulted in the conversion of starch to sucrose, and the promotion of α-amylase, β-amylase, and starch phosphorylase activities by prohexadione–calcium was superior to that of gibberellin treatment (Figure 6). The α-amylase, β-amylase, and starch phosphorylase activities under EAN and GAN treatments of No. 3 increased significantly from the 10th day to the 13th day of stress compared with N treatment by 7.28–8.22% and 2.46–6.11%, 28.37–32.92% and 18.88–22.80%, and 15.34–18.41% and 12.03–15.90% (Figure 6A,C,E). For the HHz variety, the α-amylase, β-amylase, and starch phosphorylase activities reached peak values on day 13, with significant increases of 17.80% and 11.40%, 25.24% and 38.18%, and 4.73% and 3.00%, respectively (Figure 6B,D,F).

### 3.6. Effects of Prohexadione–Calcium, Gibberellin, and NaCl Stress on the Morphological, Photosynthetic, and Carbon Metabolism Indices of Rice Seedlings of Different Varieties (F Values)

The N treatment, EAN treatment, and GAN treatment had significant effects on the morphology, photosynthesis, and carbon metabolism of the No. 3 variety rice seedlings. For the HHz variety, the N treatment had significant effects on all parameters except dry weight; while the EAN and GAN treatments had significant effects on the morphological, photosynthetic, and carbon metabolic pathways of the HHz variety (Supplementary Table S1). The interaction between the NaCl stress time and both the EAN and GAN treatments had significant or extremely significant effects on the morphological, photosynthetic, and carbon metabolic pathways of rice seedlings, respectively. The results showed that the leaf spraying of EA and GA had positive effects on the growth and physiological properties of rice seedlings under different NaCl stress times.

### 3.7. Related Analysis

**Figure 7:**
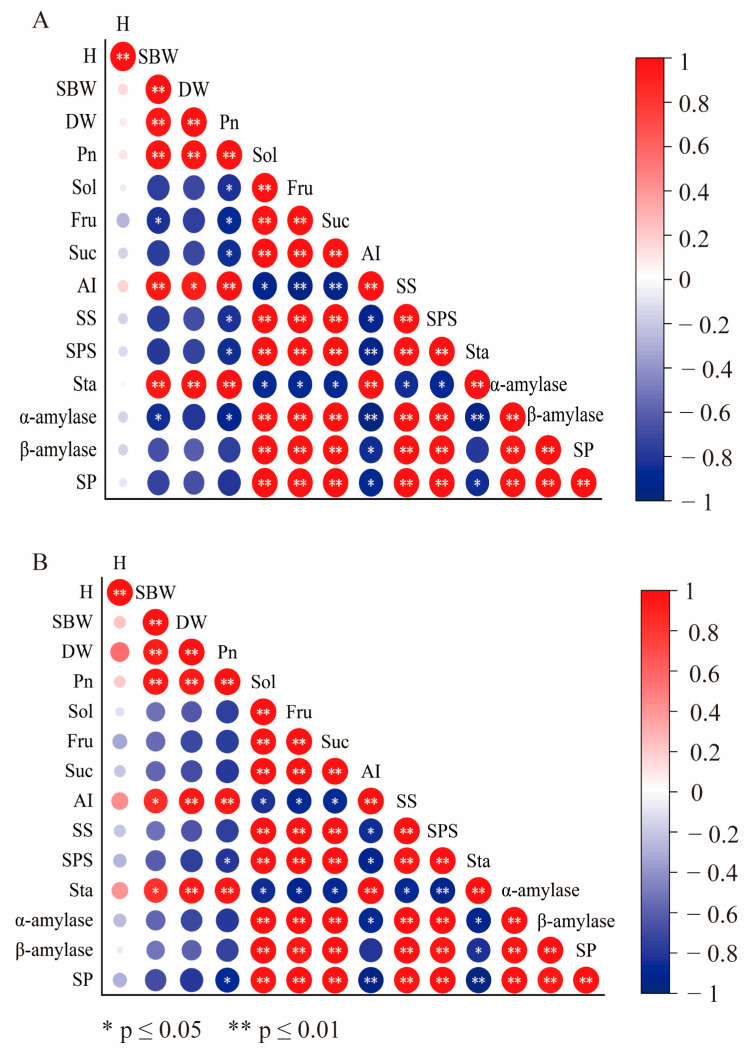
Correlation analysis between different indices for the No. 3 and HHz rice seedling varieties under NaCl stress with prohexadione–calcium and gibberellin treatments. (**A**) refer to No. 3; (**B**) refer HHz. Abbreviations: H, plant height; SBW, stem base width; DW, dry weight; Pn, net photosynthetic rate; AI, acid convertase; Suc, sucrose; Sol, soluble sugar; Fru, fructose; α-amylase; β-amylase; SS, sucrose synthase; SPS, sucrose phosphate synthase; SP, starch phosphorylase; Sta, starch.

Pearson’s correlation analysis was performed to examine the correlations between 14 representative traits (Figure 7). The dry weight of No. 3 was positively correlated with the stem base width, Pn, and starch. Sucrose was positively correlated with SS and SPS and negatively correlated with AI and Pn. Starch was positively correlated with Pn and negatively correlated with sucrose, soluble sugar, fructose, starch phosphorylase, and α-amylase (Figure 7A). There were significant positive correlations between the dry weight of HHz and the stem base width, Pn, and starch. Sucrose was negatively correlated with AI and positively correlated with SS and SPS. Starch was positively correlated with Pn and negatively correlated with sucrose, soluble sugars, fructose, α-amylase, β-amylase, and starch phosphorylase (Figure 7B).

**Figure 8:**
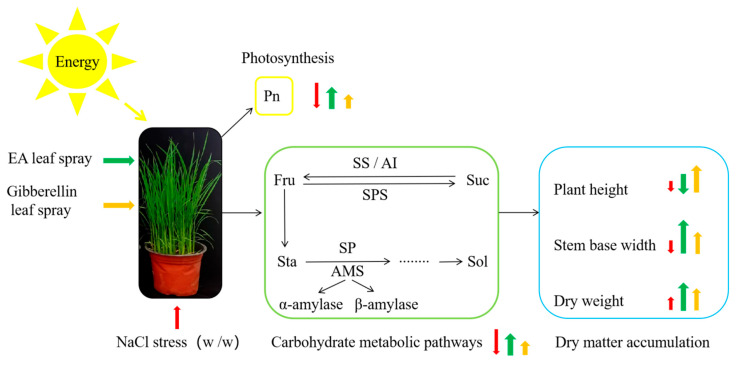
Effects of leaf spraying with prohexadione–calcium and gibberellin on the morphogenesis, photosynthesis, and carbohydrate metabolism of rice seedlings under NaCl stress. The red arrow represents the effect of NaCl stress; the green arrow represents the effect of prohexadione–calcium + NaCl treatment; and the yellow arrow represents the effect of gibberellin + NaCl treatment. The sizes of the different colored arrows represent the promoting or inhibiting effects of the different treatments on the different indices of rice seedlings. Pn, net photosynthetic rate; AI, acid convertase; Suc, sucrose; Sol, soluble sugar; Fru, fructose; SS, sucrose synthase; SPS, sucrose phosphate synthase; SP, starch phosphorylase; Sta, starch; AMS, amylase.

Prohexadione–calcium and gibberellin leaf spraying of rice seedlings maintained the growth of rice seedlings under NaCl stress by regulating the carbohydrate metabolic pathway of rice seedlings, enhancing the availability of photosynthetic products, and improving photosynthesis. Prohexadione–calcium was better than gibberellin treatment (Figure 8).

## 4. Discussion

### 4.1. Effects of EA and GA on the Phenotype of Rice Seedlings Under Salt Stress

Salt stress inhibits plant growth by increasing osmotic stress [18]. In this experiment, the seedlings of No. 3 and HHz rice varieties grew slowly under NaCl stress. The rate of dry matter accumulation gradually reduced (Figure 2) and the cells had a lower water potential. This may be due to the reduction in carbon assimilation caused by NaCl stress. Previous studies have claimed that prohexadione–calcium reduces plant height by inhibiting the synthesis of gibberellin [26], and GA can alleviate the growth inhibition effects of salt stress on plants [27]. In this study, the effects of EA and GA on the height of rice seedlings under salt stress were in the opposite direction (Figure 2A,B). Compared with the N treatment, the EAN treatment significantly reduced the height of rice seedlings and increased the stem base width and dry matter accumulation (Figure 2A–F), which can improve the plant’s survival at the later transplanting stages. GAN treatment alleviated the limitation of NaCl stress on rice seedling height growth (Figure 2A,B) and had a positive effect on promoting material accumulation (Figure 2C–F). This is consistent with the reported effect of GA on the dry weight of seedlings under salt stress [28]. On the whole, EA reduced the internode length of rice seedlings and increased the stem width, while GA increased the internode length of rice seedlings to alleviate the growth inhibition effect of NaCl stress.

### 4.2. Effects of EA and GA on Photosynthesis and Sucrose Metabolism-Related Products in Rice Seedlings Under Salt Stress

Salt stress reduces the water potential of cells and their photosynthetic rate, thereby reducing carbon assimilation and energy supply and inhibiting plant growth [29]. Photosynthesis is the most important process in plant growth and development [30] and the basis for material accumulation [31,32]. In this study, it was found that both EA and GA treatments had a better promotion effect on the Pn of rice seedlings under salt stress, and the effect of EA treatment was better than that of GA treatment (Figure 3). The Pn of EAN and GAN treatments was significantly higher from day 10 to 13 compared with the N treatment (Figure 3), which was favorable for rice seedlings to accumulate more assimilated products for growth and development under NaCl stress. These results are similar to those of previous studies on the effects of plant growth regulators on photosynthetic changes in rice under NaCl stress [33]. The major components of the carbon metabolic pathway are starch and sugar, with starch as the carbon source and sugar as the intermediate product [34]. The main product of photosynthesis is sucrose [35], and sucrose accumulation is the mechanism underlying the adaptation of rice to salt stress [36]. SPS is one of the key enzymes involved in sucrose synthesis [37,38]. A decrease in AI activity has little effect on sucrose [2], while SS plays a major role in sucrose decomposition [37]. One of the decomposition products is fructose, which can be used as a substrate for starch and other carbohydrate biosynthesis processes [39]. In this study, the sucrose and fructose contents increased significantly with the prolongation of the NaCl stress time (Figure 4C–F). This may be an adaptive response of rice seedlings to NaCl stress or the result of carbon utilization. The accumulation of carbohydrates, such as sucrose, facilitates the maintenance of cell expansion pressure in rice seedlings while maintaining the stability of cell membranes. This is consistent with previous studies on the effect of salt stress on rice [36]. Sucrose was positively correlated with SPS and SS and negatively correlated with AI (Figure 7). This suggests that enhanced SPS activity leads to an increased sucrose synthesis rate, NaCl stress inhibits AI activity, and SS plays a dominant role in sucrose decomposition (Figure 5A–F). One of the pathways involved in the improved salt tolerance of plants is osmoregulation [20], and the accumulation of sugar can promote improvements in salt tolerance [40,41]. In the current study, the EA and GA treatments significantly increased the SS and SPS activity of rice seedlings under NaCl stress (Figure 5C–F), with high accumulation of sucrose and fructose (Figure 4C–F). It indicates that the application of EA and GA increases the content of osmoregulatory substances such as sugars by increasing the rate of sucrose catabolism and synthesis such that rice seedlings can accumulate more reserve substances under NaCl stress. The SS, SPS, and AI activities were stronger under NaCl stress, and the regulation of the sucrose metabolism pathway was better under NaCl stress, which is beneficial for reducing water loss and maintaining basal metabolism.

### 4.3. Effects of EA and GA on Products Related to Photosynthesis and Starch Metabolism in Rice Seedlings Under Salt Stress

Starch accumulates soluble sugars under abiotic stress to maintain the basal metabolic balance of plants [25]. Amylase catalyzes the starch production of maltose and glucose [4]. Increased α-amylase activity facilitates the formation of more soluble sugars, resulting in better osmotic protection [42]. Increased starch phosphorylase activity under salt stress contributes to starch degradation and mobilizes sugars. Salt stress inhibits the conversion of some soluble sugars to starch [43]. The present study found that the starch content of rice seedlings decreased with increasing NaCl stress time (Figure 4G,H), while the soluble sugar content and starch phosphorylase activity showed the opposite trend (Figure 4A,B and Figure 6E,F). These results indicate that NaCl stress promotes the transformation of starch to sugar, increases the accumulation of photosynthate, and enhances the osmotic protection ability and rapid energy supply of rice seedlings. The decrease in the starch content may be due to the insufficient synthesis of carbon sources [2]. In the current study, we found that the α-amylase, β-amylase, and starch phosphorylase activities were significantly increased under NaCl stress (Figure 6), and the sucrose and starch contents were also increased (Figure 4C,D,G,H). These results indicate that EA and GA enhance the rate of starch metabolism, which may be one of the reasons for the increase in sucrose content. Although the β-amylase activity of HHz GAN treatment was significantly higher than that of EAN treatment at the 13th day of NaCl stress, the combined effect of prohexadione–calcium treatment to increase the activity of enzymes related to the starch metabolic pathway was better than that of gibberellin treatment. These results indicate that EA can better enhance the carbon energy storage and survival abilities of rice seedlings under NaCl stress.

## 5. Conclusions

In the current study, NaCl stress inhibited the growth and photosynthesis rate of rice seedlings and destroyed the carbon metabolism balance. EA and GA leaf spraying under NaCl stress enhanced the growth rate of rice seedlings and increased photosynthesis, providing a carbon source for the carbon metabolic pathway (Figure 8). Under NaCl stress, EA and GA leaf spraying enhanced starch decomposition by increasing starch phosphorylase activity, increased the contents of sucrose and soluble sugars, and enhanced the osmotic adjustment ability of rice seedlings under NaCl stress. The sucrose synthase and sucrose phosphate synthase activities were enhanced, which increased the rate of sucrose decomposition and synthesis and provided substrates for other metabolic processes. Therefore, prohexadione–calcium and gibberellin leaf spraying were more effective in maintaining the balance of carbon metabolic pathways in rice seedlings under NaCl stress by enhancing material accumulation and photosynthesis. Overall, it appeared that prohexadione–calcium was more effective in regulating the carbohydrate pathway of rice seedlings and relieving the growth inhibition of rice seedlings by NaCl stress.

## Data Availability

Data are contained within the article.

## Supplementary Materials

The following supporting information can be downloaded at https://www.mdpi.com/article/10.3390/plants14081240/s1. Table S1: Interaction between treatments (F value).
