# Supplementary material for: Comparative Study of the Mechanisms Underlying the Effects of Prohexadione-Calcium and Gibberellin on the Morphogenesis and Carbon Metabolism of Rice Seedlings Under NaCl Stress
Source: Plants (Basel). 2025 Apr 18;14(8):1240. doi: 10.3390/plants14081240 (PMC12030018; doi:10.3390/plants14081240)
Supplement: Supplementary file 1 [file plants-14-01240-s001.zip › plants-3550783-supplementary.pdf]

Supplementary Table 1 Interaction between treatments (F value)

		H	SBW	DW	Pn	Fru	Sol	Sta
No.3	A	130.13**	210.13**	251.58**	546.19**	4228.35**	782.84**	3977.08**
	A * B	13.44**	0.46	2.60	53.77**	178.55**	79.55**	360.97**
	C	10.01**	864.90**	538.87**	150.54**	551.14**	530.69**	726.04**
	C * B	0.48	20.37**	6.37**	13.99**	2.80	13.07**	31.94**
	D	3376.96**	216.60**	695.69**	379.10**	84.70**	222.66**	134.94**
	D * B	264.58**	13.04**	32.00**	26.04**	0.82	10.79**	5.64**
		Suc	AI	SS	SPS	α-amylase	β-amylase	SP
No.3	A	397.93**	2786.06**	2103.60**	400.20**	2368.49**	2733.32**	1343.00**
	A * B	50.98**	285.31**	204.73**	34.52**	326.02**	77.10**	88.58**
	C	265.09**	72.00**	314.39**	426.22**	180.87**	1156.92**	784.98**
	C * B	18.25**	6.99**	12.68**	29.82**	4.75**	12.86**	86.33**
	D	123.81**	22.69**	129.24**	43.33**	104.17**	158.38**	312.88**
	D * B	22.88**	0.79	4.98*	10.37**	5.63**	25.92**	67.83**
		H	SBW	DW	Pn	Fru	Sol	Sta
HHZ	A	358.04**	17.07**	1.82	363.92**	3368.78**	283.45**	4951.26**
	A * B	46.62**	0.36	1.26	94.62**	244.26**	27.24**	474.15**
	C	49.27**	310.08**	1352.15**	97.54**	369.74**	11.03**	625.12**
	C * B	20.57**	30.31**	83.90**	17.16**	3.44*	0.25	1.53
	D	10593.35**	101.40**	118.38**	19.65**	66.41**	219.43**	1344.50**
	D * B	320.63**	9.67**	8.02**	4.46*	18.81**	3.89*	19.10**
		Suc	AI	SS	SPS	α-amylase	β-amylase	SP
HHZ	A	576.75**	607.94**	1694.11**	123.54**	3317.78**	3727.89**	2127.53**
	A * B	44.84**	78.03**	121.16**	43.69**	519.39**	143.96**	234.50**
	C	169.35**	34.17**	470.09**	296.73**	789.86**	1360.61**	197.03**
	C * B	14.45**	4.02*	28.19**	13.27**	44.61**	6.39**	3.98*
	D	167.71**	25.35**	259.05**	125.24**	1020.55**	2246.88**	36.58**
	D * B	12.69**	6.93**	13.16**	12.43**	45.94**	135.29**	1.02

A: NaCl treatment; **B**: Processing time; **C**: ‘Prohexadione-calcium+NaCl treatment’(EAN); **D**: ‘Gibberellin+NaCl treatment’(GAN); H: Plant height; SBW: Stem base width; DW：

Dry weight；Pn: Net photosynthetic rate; Fru: Fructose; Sol: Soluble sugar; Sta: Starch; Suc: Sucrose; AI: Acid convertase; SS: Sucrose synthase; SPS: Sucrose phosphate synthase; α-amylase; β-amylase; SP: Starch phosphorylase.
